# Reduced levels of alpha-1-antitrypsin in cerebrospinal fluid of amyotrophic lateral sclerosis patients: a novel approach for a potential treatment

**DOI:** 10.1186/s12974-016-0589-4

**Published:** 2016-06-01

**Authors:** Uri Wormser, Jessica Mandrioli, Marco Vinceti, Nicola Fini, Amnon Sintov, Berta Brodsky, Elena Proskura, Yoram Finkelstein

**Affiliations:** Institute of Drug Research, School of Pharmacy, Faculty of Medicine, The Hebrew University, POB 12065, 91120 Jerusalem, Israel; Department of Neuroscience, S. Agostino- Estense Hospital, and University of Modena and Reggio Emilia, Modena, Italy; Research Centre for Environmental, Genetic and Nutritional Epidemiology (CREAGEN), University of Modena and Reggio Emilia, Modena, Italy; Department of Biomedical Engineering, Faculty of Engineering Sciences, Ben Gurion University of the Negev, Beer Sheva, 84105 Israel; Service and Unit of Neurology and Toxicology, Shaare Zedek Medical Center, Jerusalem, Israel; David R. Bloom Center for Pharmacy at the Hebrew University and, The Dr. Adolf and Klara Brettler Centre for Research in Molecular Pharmacology and Therapeutics at the Hebrew University, Jerusalem, Israel

**Keywords:** Antitrypsin, Amyotrophic lateral sclerosis, Motor neuron disease, Cerebrospinal fluid, ALS, MND

## Abstract

**Background:**

Amyotrophic lateral sclerosis (ALS) is an incurable neurodegenerative motor neuron disease that involves activation of the immune system and inflammatory response in the nervous system. Reduced level of the immuno-modulatory and anti-inflammatory protein alpha-1-antitrypsin (AAT) is associated with inflammation-related pathologies. The objective of the present is to determine AAT levels and IL-23 in the cerebrospinal fluid (CSF) of ALS patients and control group.

**Findings:**

CSF samples from newly diagnosed ALS patients and age-matched controls were analyzed for AAT and IL-23 by ELISA and magnetic luminex screening, respectively. A statistically significant reduction of 45 % in mean AAT levels was observed in the CSF of ALS patients (21.4 μg/ml) as compared to the control group (mean 38.8 μg/ml, *p* = 0.013). A statistically significant increase of 30.8 % in CSF mean levels of the pro-inflammatory cytokine IL-23 was observed in ALS patients (1647 pg/ml) in comparison to the controls (1259 pg/ml, *p* = 0.012). A negative correlation coefficient (*r* = −0.543) was obtained by linear regression analysis of the two measured parameters (*p* = 0.036).

**Conclusions:**

Reduced AAT and elevated IL-23 CSF levels support the notion of neuroinflammatory process occurring in ALS patients. Increasing AAT levels in the patients’ nervous system should be further investigated as a new therapeutic approach and a novel potential tool for ALS treatment.

## Findings

### Background

Amyotrophic lateral sclerosis (ALS) is an incurable, neurodegenerative motor neuron disease with devastating impact that relentlessly causes injury and death of the lower motor neurons in the brainstem and spinal cord and of the upper motor neurons in the motor cortex [1]. Patients experience signs and symptoms of progressive muscle atrophy and weakness, fatigue and dysphagia, and typically succumb to respiratory failure [2, 3]. The median survival period from the clinical onset to the fatal outcome ranges from 20 to 48 months. However, a relatively small group (10–20 %) of the patients survives more than 10 years [4]. ALS is most commonly a sporadic disease while 10–15 % of cases are familial [5]. The worldwide incidence of ALS is approximately 2–3 per 100,000 individuals [1], except for a few high-incidence foci, such as the Kii Peninsula and Guam [6].

Accumulating data support the notion that inflammatory processes are involved in ALS pathophysiology. Activation of microglia and astrocytes was demonstrated in both animal model and humans. Brain infiltration of both CD4^+^ and CD8^+^ T lymphocytes was shown in ALS patients [7]. Regulatory T lymphocytes number was found to be a prognostic factor both in animal models of ALS and in human patients affected by the disease [8]. Alterations in pro- and anti-inflammatory cytokines and growth factors such as IL-6, IL-10, GM-CSF, G-CSF, IL-2, IL-4, IL-8, IL12, IL-15, IL-17, IL-18, basic FGF, VEGF, and IFN-gamma were observed in CSF of ALS patients [9–12].

Apart from cytokines and growth factors, proteinase inhibitors play a central role in controlling inflammation. For instance, the serine proteinase inhibitor (serpin) alpha-1-antitrypsin (AAT) blocks neutrophil elastase and prevents pathological tissue disruption. Thus, its absence may lead to certain lung pathologies such as pulmonary emphysema, cystic fibrosis, and chronic obstructive lung disease [13]. In addition, the immuno-modulatory activities of AAT is expressed by controlling proinflammatory cytokine release [14], binding to complement C3 [15] and neutrophil functions [16]. Due to its anti-inflammatory properties, AAT is a drug candidate for various disorders including panniculitis, diabetes mellitus, rheumatoid arthritis, vasculitis, and fibromyalgia [13]. In view of the key function of AAT in controlling immunological response and inflammation, this study was aimed to quantitatively determine the levels of this protein and for comparing the pro-inflammatory cytokine IL-23, in CSF specimens of ALS patients and control group.

### Subjects and methods

#### Study participants

ALS patients were recruited from a case series of residents of the Emilia-Romagna region, northern Italy. They were diagnosed with definite or probable ALS (revised El Escorial criteria) at the ALS Center of the Modena University Neurological Department from 2001 to 2011, and underwent lumbar puncture due to diagnostic procedures. This group included 58 consecutive patients with sporadic ALS; only 15 of them had at least 1 ml CSF available for the present study. The 15 patients (10 males, 5 females) had at onset a mean age of 52 years (range 31–62 years); 13 patients presented with spinal onset and 2 with bulbar onset ALS. No patient presented with ALS-associated dementia or extrapyramidal signs. Distributions of phenotypes were as follows: 8 classic, 3 flail, 2 bulbar, and 2 upper motor neuron predominant phenotype. Mean disease duration from onset to death or last observation was 67 months. All the patients started Riluzole treatment after CSF sample withdrawal. Ten of them underwent genetic screening including all the major genes that are implicated in ALS (SOD1, C9ORF72, FUS, TDP43) with none testing positive. Genetic analysis could not be performed in the remaining patients due to lack of available blood samples.

The control population consisted of residents in the Emilia-Romagna region who were admitted to the same department between 2001 and 2011, and underwent lumbar puncture because of suspected but later on unconfirmed neurological disease. The signs and symptoms that led to neurological examination and lumbar puncture were mainly headache and paresthesia. Fourteen of these subjects were randomly selected and age matched (±10 yrs) 1:1 to ALS patients. The 14 age-matched controls had a mean age of 54 years (range 32–71 years). All the control patients were subsequently discharged from hospital without a diagnosis of a major disease.

Informed consent for diagnostic lumbar puncture (LP) was obtained from all the patients, and utilization of the CSF specimens for the present study was approved by the Modena Ethical Committee.

#### Sample collection

Approx. 6–8 mL of CSF were collected by LP from each patient and immediately stored at −80 °C until in polypropylene tubes. A 1 ml aliquot was transported by air courier, deep frozen in dry ice to the Institute of Drug Research (U. W.’s laboratory) at the Hebrew University of Jerusalem, and kept continuously frozen until use.

#### Determination of AAT and IL-23

AAT was determined (monoplicate) by ELISA kit validated for CSF (Assaypro, St. Charles, MO, USA, Cat No. EA5101-1) and cytokines by Human Magnetic Luminex screening assay (R&D Systems, Minneapolis, MN, USA).

#### Data analysis

Statistical significance of the difference between the ALS and control groups was calculated by Student’s *t* test. Correlation coefficient of AAT and IL-23 data in ALS patients was calculated by Pearson’s analysis.

## Results and discussion

**Fig. 1:**
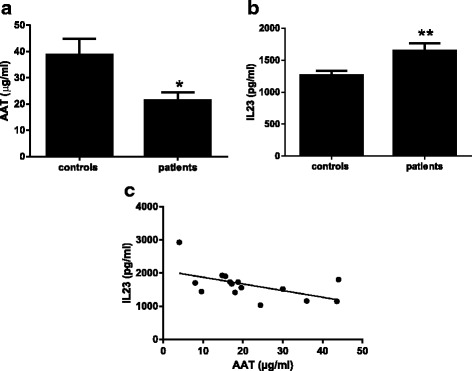
AAT and IL-23 levels in CSF of ALS patients and controls. CSF specimens of patients and controls were determined for AAT (**a**) and IL-23 (**b**) by ELISA kit and Human Magnetic Luminex screening assay, respectively. Results are expressed as mean ± SEM using Student’s *t* test for calculating the statistical significance of the difference between the two tested groups. *N* = 15 for both AAT and IL-23 in ALS group while in the controls *n* = 14 for AAT and *n* = 13 for IL-23 determinations. *p = 0.014, **p = 0.012; **c** Linear regression (Pearson test) of AAT and IL-23 in ALS patients. *R* = −0.544; *p* = 0.036

The present study demonstrates a statistically significant reduction of 45 % in AAT mean CSF levels in ALS patients (21.4 μg/ml) as compared to the control group (mean 38.8 μg/ml, *p* = 0.014) (Fig. 1a, Table 1). The median values showed even greater reduction of 53 % in AAT levels in the patients (18 μg/ml) in comparison to the controls (38 μg/ml) (Table 1). These values are comparable to those previously found in human CSF by other laboratories [17–19]. In line with these findings, a statistically significant increase of 30.8 % in CSF mean levels of the pro-inflammatory cytokine IL-23 was observed in ALS patients (1647 pg/ml) in comparison to the controls (1259 pg/ml, *p* = 0.012) (Fig. 1b, Table 1). The median value showed 36 % increase in the patients group (1678 pg/ml) as compared to the controls (1229 pg/ml) (Table 1). No statistically significant differences between both groups were detected for the other cytokines (IL-8, MCP-1, IL-17A, growth-related oncogene-alpha, unpublished data). As expected, a negative correlation coefficient (*r* = −0.544) was obtained by linear regression analysis of the two measured parameters in ALS patients (*p* = 0.036) (Fig. 1c). No linear correlation was observed between AAT and IL-23 in the control group (*r* = 0.065) (linear regression data not shown).

**Table 1:** Quantification of AAT and IL-23 in CSF of ALS patients and controls

Tested parameter	Tested group	Mean ± SEM	Change^a^ (%)	Median	Change^a^ (%)
AAT	Control	38.8 ± 6.0 μg/ml		38.0 μg/ml	
AAT	Patient	21.4 ± 3.1 μg/ml*	−45	18.0 μg/ml	−53
IL-23	Control	1259 pg/ml		1229 pg/ml	
IL-23	Patient	1647 pg/ml**	+30.8	1678 pg/ml	+36

CSF specimens of patients and controls were determined for AAT and IL-23 by ELISA kit and Human Magnetic Luminex screening assay, respectively. Results are expressed as median or mean ± SEM using Student’s *t* test for calculating the statistical significance of the difference between the two tested groups. *N* = 15 for both AAT and IL-23 in ALS group while in the controls *n* = 14 for AAT and *n* = 13 for IL-23 determinations

*p = 0.014, **p = 0.012

^a^Expresses the percent of change (reduction or elevation) taking the control as 100 %

To our best knowledge, this is the first report on a significant reduction of AAT levels in CSF of ALS patients. The lack of this anti-inflammatory and immune-modulatory protein is inversely correlated with the increase of the pro-inflammatory cytokine IL-23 in the patients. These findings are compatible with the accumulating evidence for the link between ALS and inflammation [7, 20] expressed by alterations in levels of cytokines and growth factors [9, 10, 21].

Brettschneider and colleagues through proteomic analysis identified two proteins that were upregulated (alpha-1-antitrypsin precursor and Zn-alpha-2-glycoprotein) and three proteins (ceruloplasmin precursor, transferrin, and beta-2-microglobulin) that were downregulated in the CSF of ALS patients [22]. Elevation of AAT precursor and reduction in AAT levels may stem from inappropriate conversion of the precursor to its final product leading to decrease in AAT levels in CSF of ALS patients. Another study identified serine proteases and serpins (alpha-1-anti-trypsin, alpha-1-anti-chymotrypsin, and protease nexin I) in motoneurons in neurofilament conglomerates suggesting that the imbalance of serine proteases and internalized serpins may play a role in the pathogenesis of ALS [23].

Whether altered AAT levels are a result of the pathological processes in the neuronal system or a factor involved in the evolution of the disease remains an open question. Nevertheless, the central role of AAT in controlling various inflammatory processes, and the variety of diseases associated with inappropriate levels of AAT may lead to the notion that lack of this protein may be involved in the pathogenic process of ALS or in its clinical course. Should these results be confirmed in larger studies, increasing levels of this protein in the neuronal system of the patients may serve as a novel therapeutic approach for ALS. Replacement therapy by intravenous augmentation of AAT has been approved by the US-FDA for AAT deficiency such as in individuals with severe COPD or emphysema [24]. However, targeting the CNS is more convoluted and challenging and needs an alternative route of administration. As it is evident now that a direct pathway connects a submucosal compartment in the nasal passages to brain interstitial fluid, the nose-to-brain delivery of large molecular weight molecules can be feasible via intranasal administration. Thus, the central nervous system accessibility can be achieved by intranasal administration of a nanotechnology-based compounded protein [25], which may also be a convenient and efficient procedure for ALS patients.

## Abbreviations

AAT, alpha-1-antitrypsin; ALS, amyotrophic lateral sclerosis; COPD, chronic obstructive pulmonary disorder; CSF, cerebrospinal fluid; IL-23, interleukin-23
